# The Efficacy of Using Patient-Derived Organoids to Predict Treatment Response in Colorectal Cancer

**DOI:** 10.3390/cancers15030805

**Published:** 2023-01-28

**Authors:** Chang Su, Kelly A. Olsen, Catherine E. Bond, Vicki L. J. Whitehall

**Affiliations:** 1 Conjoint Gastroenterology Laboratory, QIMR Berghofer Medical Research Institute, Herston 4006, Australia; 2 Faculty of Medicine, The University of Queensland, Herston 4006, Australia; 3 Conjoint Internal Medicine Laboratory, Pathology Queensland, Herston 4006, Australia

**Keywords:** colorectal cancer, organoids, personalised medicine

## Abstract

**Simple Summary:**

Colorectal cancer is the third most commonly diagnosed cancer. Patients may receive chemotherapy, targeted therapy, immunotherapy, radiotherapy, and/or surgery. However, not all patients will benefit from each available treatment option. There has been rising interest in personalised medicine, which refers to tailoring treatment plans to the unique characteristics of each patient’s cancer. Patient-derived tumour organoids are three-dimensional models of patients’ cancer cells which can be tested for sensitivity to various treatment options in the laboratory. This review summarises studies which have explored whether the organoid’s sensitivity in the laboratory corresponds to the patient’s response in the clinic. Tumour organoids are promising models for personalised medicine in the context of selecting chemotherapy and radiotherapy options. Further advancements in organoid technology are required for testing immunotherapy and certain targeted therapy options. Overall, future clinical trials of organoid testing prior to treatment commencement will support the implementation of organoid-based personalised medicine in the clinic.

**Abstract:**

Colorectal cancer is an important cause of morbidity and mortality worldwide. The current treatment landscape includes chemotherapy, targeted therapy, immunotherapy, radiotherapy, and surgery. A key challenge to improving patient outcomes is the significant inter-patient heterogeneity in treatment response. Tumour organoids derived from the patients’ tumours via surgically resected or endoscopically biopsied tissue, have emerged as promising models for personalised medicine. This review synthesises the findings, to date, of studies which have explored the efficacy of ex vivo organoid sensitivity testing for predicting treatment response. Most studies have focused on predicting the response to standard-of-care radiotherapy and chemotherapy options. There is strong evidence to support organoid sensitivity testing of ionising radiation, 5-fluorouracil, and irinotecan, and to a lesser extent, oxaliplatin and TAS-102. Fewer studies have used organoids to identify patients who are likely to benefit from novel treatment options that otherwise remain in clinical trials. This review also summarises recent advancements in organoid culture to include non-epithelial components of the tumour microenvironment, to allow testing of immunotherapy and certain targeted therapy options. Overall, further prospective trials will support the implementation of organoid-based personalised medicine for colorectal cancer patients in the future.

## 1. Introduction

Colorectal cancer (CRC) is the third most commonly diagnosed cancer worldwide [1]. Surgical resection is the mainstay of curative treatment for patients with localised disease. Adjuvant chemotherapy with 5-fluorouracil (5FU), often in combination with oxaliplatin, is indicated following resection in patients with stage III disease or high-risk stage II disease [2]. Additionally, neoadjuvant chemoradiotherapy may be recommended prior to resection in patients with rectal cancer [3].

Approximately 20–50% of patients presenting with localised CRC will progress to stage IV metastatic disease following initial treatment [4]. In patients with unresectable metastases, palliative chemotherapy with 5FU in combination with oxaliplatin or irinotecan, becomes first-line treatment [5]. Further systemic treatment options include anti-epidermal growth factor receptor (EGFR) antibodies, kinase inhibitors, anti-angiogenic agents, and immunotherapy agents [5]. Despite these treatment options, the median overall survival of patients with metastatic CRC is only 30 months [5]. There is significant inter-patient heterogeneity in the response to treatment [6], which presents a key challenge in improving patient outcomes.

Despite enormous genomic sequencing efforts, few predictive biomarkers exist to inform patient regimens. Activating *KRAS* mutations, which are present in 40% of CRCs, confer resistance to anti-EGFR therapy via constitutive activation of the downstream mitogen-activated protein kinase (MAPK) pathway [7]. The combination of cetuximab (anti-EGFR antibody) and encorafenib (BRAF V600E inhibitor) has recently emerged as a line of treatment for metastatic *BRAF* V600E-mutant cases [8]. Additionally, deficient mismatch repair, which is present in 15% of CRCs, is predictive of sensitivity to immunotherapy due to an increased neoantigen burden [9,10]. Deficient mismatch repair is also predictive of resistance to 5FU chemotherapy in the adjuvant setting [11]. However, molecular biomarkers are not fully predictive of patient response and not available for all treatment options, highlighting the potential role of personalised medicine in further addressing inter-patient heterogeneity.

Personalised medicine involves tailoring treatment to the unique characteristics of each patient’s cancer. This requires the use of time- and cost-efficient patient-derived models to predict which treatment options are most likely to be effective [12]. Treatment options unlikely to be of benefit may also be predicted, which can prevent unnecessary exposure to treatment-emergent adverse events. There is rising interest in the role of patient-derived tumour organoids. Patient-derived tumour organoids are three-dimensional, self-organising cultures of tumour cells isolated from surgical or endoscopic tissue samples [13]. They have extensively been shown to recapitulate the characteristics and heterogeneity of the original patient tumour, and therefore have increasingly been used as models in drug mechanism and sensitivity studies [14,15,16,17]. Here, we review studies that have explored the relationship between ex vivo organoid sensitivity and patient treatment response, to evaluate the role of patient-derived tumour organoids in personalised medicine for CRC.

## 2. Methods Used in Studies of Organoid Drug Sensitivity and Patient Treatment Response

### 2.1. Colorectal Cancer Organoid Establishment and Culture

Patient-derived organoids were first generated from normal and cancerous colon tissue containing LGR5+ stem cells [13]. Protocols to establish CRC organoids from surgical or endoscopic tissue samples vary between research groups. Briefly, the tissue sample is excised and divided into a small 1 to 3 mm^3^ fragment using a scalpel blade. These tissue fragments are enzymatically digested into single cells or small clumps, which are resuspended in a basement membrane extract such as Matrigel, Geltrex, or Cultrex [18,19]. Patient-derived LGR5+ adult stem cells within the matrix are supplemented with a culture medium containing niche factors that facilitate self-renewal and epithelial differentiation. Consequently, the stem cells grow into polarised epithelial structures with differentiated villus compartments, forming the organoids [13]. Key niche factors include Wnt pathway agonists (Wnt-3a and R-spondin 1), TGFβ pathway inhibitors (A83-01 and Noggin), p38 MAPK inhibitor (SB202190), and epidermal growth factor (EGF) [13]. Media formulations used for CRC organoid culture in drug sensitivity studies are summarised in Supplementary Table S1. The efficiency of establishing organoid cultures from patient tumour tissue in these studies has frequently been reported as 70% or greater [20,21,22,23,24,25,26].

### 2.2. Patient-Derived Tumour Organoids Molecularly Recapitulate the Original Tumour

Patient-derived tumour organoids can accurately represent the genomic landscape of their tissue of origin. High rates of mutational overlap at 96% have been reported between corresponding organoids and their original tumour tissue [27], and very similar median mutation rates of 3.7 and 3.6 per Mb, respectively, have been found [14]. Global DNA methylation patterns and gene expression signatures at a single cell level have been maintained between tumour organoids and matching tissue [28]. Additionally, copy number variations (CNVs) have been reported to correspond closely [14,28], particularly if conditioned media rather than chemically defined media are used and if cultures are maintained for shorter periods of time [28]. The high degree of genetic correlation between tumour organoids and their derived tissue indicates that tumour organoids are a highly relevant tool in research and clinical trials. A concordance of up to 90% between their in vitro drug response and patient outcomes highlights their potential in transforming predictive drug screening and supports their utilisation in the clinical setting [25,27,29].

### 2.3. Ex Vivo Organoid Sensitivity Testing

A number of research groups have performed drug sensitivity testing on patient-derived CRC organoids, as discussed in the following sections. Briefly, organoids are passaged and dissociated into single cells or small clumps for plating, and subsequently exposed to drug treatment. Protocols vary between groups in terms of plating density, basement membrane extract concentration, and treatment exposure time. Treatment exposure time ranges from 3 to 24 days, and may include medium replenishment at set intervals. Some groups also allow a recovery time of up to 10 days before treatment. These differences are summarised in Supplementary Table S2. Most groups use the ATP-based CellTitre-Glo 3D cell viability assay (Promega) as the endpoint read-out. Subsequently, the half-maximal inhibitory concentration (IC50), 50% growth rate inhibitory concentration (GR50) [30], or area under the curve (AUC) can be calculated as measures of drug sensitivity.

## 3. Standard-Of-Care Systemic Therapies

Most studies examining the role of organoid sensitivity testing in predicting patient response have focused on the traditional chemotherapy options: 5FU, capecitabine, irinotecan, and oxaliplatin. Fewer studies have included the anti-EGFR antibodies, which are indicated in wild-type *KRAS* and *BRAF* cases, or the last-line treatment options, TAS-102 and regorafenib, which are indicated in the refractory metastatic setting (Table 1).

### 3.1. Evaluation of Patient Response to Treatment

Response to treatment is primarily determined using the Response Evaluation Criteria In Solid Tumours (RECIST version 1.1) [31]. Complete clinical response is defined as the disappearance of all target lesions on imaging or endoscopy. Partial response (PR) refers to at least a 30% decrease in lesion size. Progressive disease refers to at least a 20% increase in lesion size or the development of a new lesion, which forms the basis of progression-free survival time. Stable disease occurs where there is neither a sufficient decrease nor increase in lesion size to qualify for partial response or progressive disease. In studies of rectal cancer patients receiving neoadjuvant chemoradiotherapy prior to surgical resection, the American Joint Committee on Cancer (AJCC) Tumour Regression Grading (TRG) system may alternatively be used [32]. Complete pathologic response is defined as no detection of remaining tumour cells on histological analysis. Reporting in vitro response of patient-derived tumour organoids to therapeutic agents in a way that relates to clinical patient response is difficult and may vary based on the agent being tested. A major challenge in the field will be the development of standardised reporting methods to most accurately guide treatment decisions.

### 3.2. Traditional Chemotherapy

#### 3.2.1. 5-Fluorouracil

5FU is the mainstay of chemotherapy for CRC in both adjuvant and metastatic settings. As a fluoropyrimidine anti-metabolite, 5FU incorporates into tumour cell DNA and RNA, and inhibits thymidylate synthase, a nucleotide synthesis enzyme. The resulting DNA and RNA damage inhibits tumour cell proliferation and survival [33]. 5FU is administered intravenously. The orally administered 5FU prodrug, capecitabine, may alternatively be used [34]. 5FU is often used in combination with irinotecan (FOLFIRI) or oxaliplatin (FOLFOX), particularly in the metastatic setting. Hence, comparing ex vivo 5FU sensitivity of patient-derived models to patient response can be complex. Additionally, the folate analogue leucovorin is administered together with 5FU, but often omitted in ex vivo testing, as it enhances the anti-tumour effect of 5FU without having a direct effect when used alone [35]. Studies investigating the role of patient-derived tumour organoids in predicting the response to traditional chemotherapy have enrolled patients receiving various combinations of 5FU, oxaliplatin, and irinotecan, and additional targeted agents.

Some studies have tested the ex vivo sensitivity to 5FU alone on tumour organoids derived from patients receiving 5FU-based combination therapy regimens [22,24,25,26,36,37]. Ganesh et al. identified a positive correlation between organoid 5FU sensitivity and the progression-free survival of rectal cancer patients after the initiation of 5FU-based chemotherapy [26]. In a study by Yao et al. of rectal cancer patients undergoing neoadjuvant chemoradiotherapy, organoid sensitivity to 5FU demonstrated 59% sensitivity and 88% specificity for identifying complete or near-complete pathologic responders [24]. In a similar study by Lv et al., the response rate was significantly higher among patients with 5FU-sensitive organoids than among patients with 5FU-resistant organoids, but there was no difference in progression-free survival [37]. These studies suggest that organoid 5FU sensitivity testing may have a role in predicting patient response, but the irinotecan and radiotherapy components within the combination treatment regimens may be confounding. Cho et al. and Hogenson et al. found that sensitivity testing for 5FU alone is also less informative in patients simultaneously receiving targeted therapy such as an anti-EGFR antibody [22,36]. For one patient in each of these studies, ex vivo resistance to 5FU was observed despite partial response to combination chemotherapy and anti-EGFR therapy. Further testing of the organoids showed sensitivity to cetuximab, which may have driven the partial responses, highlighting the importance of testing all agents within a combination treatment regimen.

In some cases it may also be informative to test the components of a treatment regimen concurrently to account for their collective effects on tumour growth, particularly if interaction effects are present. Ooft et al. measured organoid sensitivity in patients demonstrating progressive disease following FOLFIRI therapy compared with patients demonstrating partial response or stable disease [25]. There was no statistically significant difference on the testing of 5FU alone. However, organoid sensitivity to 5FU and irinotecan, when tested together, was significantly reduced in progressive disease, which demonstrated a benefit of performing concurrent testing. Cho et al. showed in patient-derived tumour organoids that 5FU treatment could enrich cancer-derived stem cells via p53-mediated Wnt pathway activation [22], demonstrating the utility of this system for providing mechanistic insight, as well as a predictive drug measurement. Given the variety of treatment regimens offered to patients with CRC, these studies highlight the complexity of implementing effective sensitivity testing on patient-derived tumour organoids.

#### 3.2.2. Irinotecan

There is strong evidence for the role of organoid testing in predicting patient response to irinotecan. Irinotecan inhibits topoisomerase I, leading to DNA damage and the arrest of DNA replication and transcription [38]. Mo et al. identified a positive correlation between organoid sensitivity to combined 5FU and irinotecan testing and progression-free survival after the initiation of FOLFIRI therapy in the metastatic setting. Similarly, as above, Ooft et al. found that organoid sensitivity to 5FU and irinotecan was reduced in patients with progressive disease following FOLFIRI therapy [25]. This study also included patients receiving second-line irinotecan monotherapy for metastatic CRC. Tumour organoids derived from patients with progressive disease demonstrated reduced sensitivity to irinotecan compared to those derived from patients with partial response or stable disease, further supporting a role for irinotecan sensitivity testing.

The use of irinotecan for colon cancer is currently limited to the metastatic setting. Clinical trials in the adjuvant setting have not recommended the use of irinotecan in combination with or in place of oxaliplatin [39,40]. However, some patients undergoing neoadjuvant chemoradiotherapy for locally advanced rectal cancer may benefit from receiving irinotecan in addition to 5FU [41]. In studies by Lv et al. and Yao et al., organoid testing of irinotecan alone showed 74–78% sensitivity and 77–79% specificity for predicting the response to neoadjuvant chemoradiotherapy regimens that included both irinotecan and 5FU [24,37]. Lv et al. also reported a significantly higher 3-year progression-free survival rate among patients with 5FU-sensitive organoids than patients with 5FU-resistant organoids [37]. These findings support the efficacy of organoid sensitivity for predicting the response to irinotecan.

#### 3.2.3. Oxaliplatin

Studies on organoid sensitivity to oxaliplatin have been less consistent. Oxaliplatin is a platinum compound that induces DNA damage [42]. Studies by Ganesh et al., Yao et al. and Mo et al. have found that organoid sensitivity to the combined testing of oxaliplatin and 5FU correlates strongly with progression-free survival of patients receiving FOLFOX [21,26,43]. Additionally, Geevimaan et al. tested organoid sensitivity to oxaliplatin alone, which achieved 70% sensitivity and 71% specificity for predicting the clinical response to FOLFOX [20]. In a further study by Wang et al., organoid testing demonstrated 63% sensitivity and 94% specificity for predicting the response in a cohort of CRC patients receiving either FOLFOX or FOLFIRI [23]. While the analysis was not stratified by the regimen or setting, the result provides some additional supporting evidence for oxaliplatin testing. Several smaller studies have also provided support [22,36,44,45,46]; however, the results of some patients in these studies may have been confounded by concurrent anti-EGFR therapy.

In contrast, studies by Narasimhan et al. and Ooft et al. found no association between organoid sensitivity and patient response to oxaliplatin. Narasimhan et al. found that combined oxaliplatin and the 5FU testing of organoids derived from peritoneal metastases failed to separate patients with progressive disease from patients with partial response or stable disease [47]. However, this study had a limited sample size of two treatment-naïve patients and two heavily pre-treated patients. Additionally, peritoneal metastases have a poor prognosis overall [48], which may have contributed to the lack of correlation with organoid sensitivity. The study by Ooft et al. also found no association between clinical response and organoid sensitivity for FOLFOX, despite identifying an association for FOLFIRI, which further challenges the ability of organoids to model response to oxaliplatin [25].

Differences in drug screening methodologies may account for the variability in findings between studies. In comparing the studies in this section, few considered the size of organoids included in the initial seeding. The size and number of organoids are crucial to ensure the reproducibility of results in subsequent viability assays [49]. To determine cell viability, most groups used Cell Titre Glo and only a few used the 3D version of the kit recommended for 3D structures. The incomplete lysis of the organoids could give rise to incorrect measurements (Supplementary Table S2). Furthermore, the chemical instability of oxaliplatin is also a potential source of uncertainty in ex vivo sensitivity testing. Oxaliplatin is susceptible to inactivation in the presence of chloride ions and penicillin G, which are common components of organoid culture media (Supplementary Table S1) [50,51], as well as dimethyl sulfoxide, which is a common solvent in drug sensitivity studies [52]. The basement membrane extract may also affect organoid sensitivity to oxaliplatin. In a recent study by Xu et al., organoids derived from liver metastases of patients with partial response or stable disease following FOLFOX therapy showed greater sensitivity than organoids derived from patients with progressive disease [53]. This finding was only apparent when the organoids were cultured on a novel collagen-conjugated hydroxypropyl cellulose allyl sponge, rather than basement membrane extract. The basement membrane extract was found to induce epithelial-mesenchymal transition in the organoids. The addition of epithelial-mesenchymal transition inhibitors to the cultures restored differential sensitivity to the oxaliplatin and 5FU combination, suggesting that basement membrane extract-induced epithelial-mesenchymal transition may limit the ability of organoid cultures to predict the response to oxaliplatin. Overall, further studies with greater consideration of methodological and patient factors are required to delineate the relationship between ex vivo organoid sensitivity and patient response.

**Table 1:** Summary of studies associating organoid sensitivity and patient response to approved systemic therapies.

Author, Year [Reference]	(Neo)Adjuvant and/or Metastatic Setting	Approved Systemic Therapies						
		5-FU or Capecitabine	Irinotecan	Oxaliplatin	Cetuximab or Panitumumab	TAS-102	Regorafenib	Bevacizumab
		Anti-Metabolite	Topoisomerase Inhibitor	Platinum Compound	Anti-EGFR	Anti-Metabolite	Tyrosine Kinase Inhibitor	Anti-VEGF
Mo et al., 2022 [43]	Metastatic	*n* = 23	*n* = 10	*n* = 13				
Hogenson et al., 2022 [36]	Metastatic	*n* = 2		*n* = 2	*n* = 2		*n* = 2	
Lv et al., 2022 [37]	Neoadjuvant	*n* = 91	*n* = 107					
Geevimaan et al., 2022 [20]	Adjuvant and Metastatic			*n* = 17				
Yao et al., 2022 [21]	Adjuvant and Metastatic	*n* = 34		*n* = 34	*n* = 3			*n* = 8
Cho et al., 2022 [22]	Metastatic	*n* = 5	*n* = 2	*n* = 2	*n* = 1			
Wang et al., 2021 [23]	Neoadjuvant and Metastatic	*n* = 66	*n* = 11	*n* = 55				
Xu et al., 2021 [53]	Metastatic	*n* = 12		*n* = 12				
Mauri et al., 2021 [46]	Metastatic	*n* = 1	*n* = 1	*n* = 1	*n* = 1			
Narasimhan et al., 2020 [47]	Metastatic	*n* = 4		*n* = 4			*n* = 1	
Arena et al., 2020 [45]	Metastatic			*n* = 3	*n* = 1			
Yao et al., 2020 [24]	Neoadjuvant	*n* = 80	*n* = 66					
Ooft et al., 2019 [25]	Metastatic	*n* = 32	*n* = 22	*n* = 10				
Ganesh et al., 2019 [26]	Neoadjuvant and Metastatic	*n* = 7		*n* = 7				
Pasch et al., 2019 [44]	Metastatic	*n* = 1		*n* = 1				
Vlachogiannis et al., 2018 [27]	Metastatic				*n* = 4	*n* = 4	*n* = 3	
	Positive association identified between organoid sensitivity and patient response							
	Potential association between organoid sensitivity and patient response							
	No association between organoid sensitivity and patient response							
	*n* = Number of patients							

### 3.3. Anti-Epidermal Growth Factor Receptor Antibodies

There are few studies correlating organoid sensitivity and patient response to the anti-EGFR antibodies, cetuximab, and panitumumab. This may be due to the existing availability of *KRAS* and *BRAF* mutation testing in guiding their use. Activating *KRAS* and *BRAF* mutations confer resistance to anti-EGFR therapy, as KRAS and BRAF are components of the MAPK pathway downstream of EGFR [7]. Combining anti-EGFR antibodies with encorafenib (BRAF V600E inhibitor) has recently emerged as a strategy to combat resistance in *BRAF* V600E cases [8]. Not all patients with a molecular profile of wild-type *KRAS* and wild-type *BRAF* will benefit from anti-EGFR therapy [54]. Hence, organoid sensitivity testing may play a role in further predicting patient response. This has been supported by several smaller studies that have included the testing of cetuximab or panitumumab [22,27,36,45,46]. One of the patients in the study by Vlachogiannis et al. had a molecular profile of *EGFR* gene amplification, wild-type *KRAS,* and wild-type *BRAF*, which would suggest susceptibility to anti-EGFR therapy [27]. However, this patient demonstrated progression when undergoing treatment with cetuximab. Accordingly, the organoids derived from this patient’s tumour showed resistance to cetuximab, which suggests a potential for tumour organoids to predict patient response to anti-EGFR therapy better than molecular biomarkers alone. Given the small sample sizes of the studies that have tested cetuximab or panitumumab, larger studies are required to further establish the associations between patient response, organoid sensitivity, and existing molecular biomarkers.

### 3.4. TAS-102

TAS-102, which is a combination of trifluridine and tipiracil, has recently been approved as a last-line treatment option for patients with refractory metastatic CRC. Trifluridine is a fluoropyrimidine anti-metabolite and acts via a similar mechanism to 5FU. Tipiracil is a thymidine phosphorylase inhibitor and inhibits the metabolism of trifluridine to maintain adequate plasma levels [55]. Despite the similarity in the mechanism to 5FU, TAS-102 remains effective in many patients with resistance to 5FU [56]. Only one study has previously correlated organoid TAS-102 sensitivity to patient response. Vlachogiannis et al. found that achieving stable disease following TAS-102 treatment rather than progressive disease was associated with increased organoid sensitivity, despite a small sample size [27]. As there are no biomarkers currently available to guide the use of TAS-102, organoid testing may have potential to predict patient response and hence warrants further investigation.

### 3.5. Regorafenib

Regorafenib is an alternative last-line treatment option for refractory metastatic CRC. It is a kinase inhibitor that targets various intracellular kinases, including BRAF and CRAF, and receptor tyrosine kinases, including vascular endothelial growth factor receptor (VEGFR), angiopoietin 1 receptor (Tie2), and fibroblast growth factor receptor (FGFR) [57]. Accordingly, regorafenib elicits anti-angiogenic effects in addition to direct anti-proliferative effects on tumour cells. Like TAS102, no biomarkers are currently available to predict patient response. In the study by Narasimham et al., only one patient received treatment with regorafenib. This patient did not respond to regorafenib, in line with the resistance of their tumour organoids on ex vivo testing [47]. In the study by Hogenson et al., two patients received regorafenib treatment [36]. The tumour organoids from the patient with progressive disease showed resistance on testing, while the tumour organoids from the patient with stable disease showed intermediate sensitivity. While the results of these studies are promising, the sample sizes are insufficient to confirm an association between organoid sensitivity and patient response.

The role of sensitivity testing for regorafenib may be limited by the inability of epithelial organoid cultures to model the anti-angiogenic mechanisms that partially underlie regorafenib’s anti-tumour effect. Three patients in the study by Vlachogiannis et al. received treatment with regorafenib. None of the tumour organoids were sensitive to regorafenib on ex vivo testing, despite the patients demonstrating varying levels of clinical response [27]. The tumour organoids were subsequently implanted as xenografts in mice. Greater reductions in xenograft blood flow and micro-vessel density following in vivo regorafenib treatment corresponded to improved clinical response. This suggests that patient-derived tumour organoids retain the ability to model response to regorafenib, but epithelial organoids alone cannot recapitulate differential anti-angiogenic effects. Further studies with larger sample sizes would determine whether epithelial organoids can recapitulate regorafenib’s direct anti-proliferative effects on tumour cells, as a component of modelling patient response.

### 3.6. Anti-Vascular Endothelial Growth Factor Agents

Anti-angiogenic agents targeting vascular endothelial growth factor (VEGF) are also used in metastatic CRC. The study by Yao et al. explored organoid testing of bevacizumab and identified a correlation with patient response [21]. However, all patients receiving bevacizumab also received FOLFOX, leading to difficulty in establishing the specific role of bevacizumab testing. Given that bevacizumab’s anti-tumour effects are predominantly driven by anti-angiogenic effects, further advancements in organoid culture to incorporate functional vascularisation are likely necessary for the optimal prediction of patient response. Various techniques have been explored, including the co-culture of organoids with endothelial cells [58]. Truelsen et al. found that bevacizumab and regorafenib inhibit endothelial cell tube formation within CRC organoids [59]. The extent of tube formation varied between organoids derived from different patients, which may reflect differences in patient response to anti-angiogenic therapy; however, clinical parameters were not measured in this study. Recent research has also focused on developing microfluidic platforms to achieve functional, perfused vasculature within organoids, which will likely allow for more comprehensive modelling of the original patient tumour [60,61].

## 4. Novel Systemic Therapies

Limited studies have explored the screening of organoids to predict the response to treatment options that otherwise remain in clinical trials for CRC (Table 2). Ooft et al. performed novel drug screening on tumour organoids derived from metastatic CRC patients receiving a final line of standard-of-care therapy following progression on one or two previous lines [62]. Based on the results of organoid screening, three patients were offered vistusertib (mammalian target of rapamycin (mTOR) inhibitor) and another three patients were offered capivasertib (Akt inhibitor). However, further disease progression was observed in all patients, except for one who demonstrated stable disease during the first two months on vistusertib monotherapy before further disease progression. Additional biopsies were obtained for organoid culture immediately prior to initiating vistusertib or capivasertib monotherapy, which confirmed ex vivo sensitivity at the time of treatment. Targeting the phosphatidylinositol 3-kinase (PI3K)-Akt-mTOR pathway has proved challenging in CRC due to the propensity for acquired treatment resistance and treatment-emergent adverse effects [63], which may contribute to the lack of patient response observed in this study.

Narasimhan et al. also performed novel drug screening on tumour organoids derived from mCRC patients. One patient was offered gemcitabine (anti-metabolite) due to sensitivity on screening, and demonstrated partial response for three months before further progression [47]. Another patient was offered vandetanib (tyrosine kinase inhibitor) based on the results of screening; however, the patient demonstrated continued progression. This study reported challenges with drug funding and access, which delayed the initiation of treatment following the organoid screening process. The time between tissue sampling and the initiation of organoid-informed treatment was six months for the patient receiving gemcitabine and 16 months for the patient receiving vandetanib, during which changes in drug sensitivity may have occurred. As with the study by Ooft et al., this study was conducted in the refractory metastatic setting involving heavily pre-treated patients. There is a need for prospective trials enrolling patients at earlier stages of treatment, to better explore the role of organoid screening in identifying tailored novel treatment options.

Additionally, two studies have reported matches between organoid sensitivity and treatment response for select patients enrolled in clinical trials of novel targeted agents for mCRC [27,36]. In the study by Vlachogiannis et al., one patient was enrolled in a phase I trial of berzosertib (ataxia telangiectasia and Rad3-related protein (ATR) inhibitor) after disease progression on multiple standard-of-care therapies [27]. This patient did not respond to VX-970 monotherapy, in line with resistance identified on ex vivo organoid testing. In the study by Hogenson et al., the tumour organoids from a patient with progressive disease following a trial of tomivosertib (MAPK interacting kinase (MNK) inhibitor) demonstrated resistance to tomivosertib on ex vivo testing. Similarly, the tumour organoids from a patient with progressive disease following a trial of bozitinib (c-Met inhibitor) demonstrated resistance to bozitinib. Another patient achieved partial response on vemurafenib (BRAF V600E inhibitor). Accordingly, this patient’s tumour organoids demonstrated sensitivity to vemurafenib on ex vivo testing, which also aligned with the presence of the *BRAF* V600E mutation. While these findings suggest a role for organoid sensitivity testing in the use of novel treatment options, they represent reports of only individual cases. The further investigation of organoid sensitivity to novel agents in larger cohorts is required, potentially as a secondary outcome in future clinical trials.

**Table 2:** Summary of studies associating organoid sensitivity and patient response to novel systemic therapies.

		Novel Systemic Therapies							
		Vistusertib	Capivasertib	Vemurafenib	Tomivosertib	Bozitinib	Gemcitabine	Vandetanib	Berzosertib
		mTOR Inhibitor	Akt Inhibitor	BRAF V600E Inhibitor	MNK Inhibitor	c-Met Inhibitor	Anti-Metabolite	Tyrosine Kinase Inhibitor	ATR Inhibitor
Hogenson et al., 2022 [36]	Metastatic			*n* = 1	*n* = 1	*n* = 1			
Ooft et al., 2021 [62]	Metastatic	*n* = 3	*n* = 3						
Narasimhan et al., 2020 [47]	Metastatic						*n* = 1	*n* = 1	
Vlachogiannis et al., 2018 [27]	Metastatic								*n* = 1
	Positive association identified between organoid sensitivity and patient response								
	Potential association between organoid sensitivity and patient response								
	No association between organoid sensitivity and patient response								
	*n* = Number of patients								

## 5. Radiotherapy

As radiotherapy is an additional treatment modality for rectal cancer, recent studies have also examined the utility of measuring the sensitivity of patient-derived tumour organoids to ionising radiation. Several studies have established a positive association between organoid radiosensitivity and patient response to radiotherapy, across both neoadjuvant and metastatic settings (Table 3) [26,64,65]. In the neoadjuvant setting, Lv et al. found that the response rate to chemoradiotherapy was significantly higher among patients with radio-sensitive organoids than among patients with radio-resistant organoids. The 3-year metastasis-free survival was significantly higher in the group with radio-sensitive organoids. The 3-year progression-free survival was also higher in this group but did not reach statistical significance. In both studies by Lv et al. and Yao et al., organoid radiosensitivity showed 42% sensitivity and 98% specificity for predicting response to neoadjuvant chemoradiotherapy [24,37]. The low sensitivity values suggest a need to also test the 5FU and irinotecan components of the chemoradiotherapy regimen. Yao et al. found that ex vivo sensitivity to at least one of the three components of the neoadjuvant chemoradiotherapy, tested individually, achieved 78% sensitivity and 92% specificity in predicting response. This suggests that the concurrent testing of components within combination treatment regimens may also be important across radiotherapy and chemotherapy modalities. Overall, the studies that have tested organoid sensitivity to ionising radiation [24,26,37,64,65] provide consistent evidence for a role of tumour organoids in predicting the response of rectal cancer to radiotherapy.

## 6. New Developments to Recapitulate the Tumour Microenvironment

Laboratory and clinical trials have shown promising predictive potential of organoid-based assays for standard-of-care therapies (Table 1). This varies somewhat between different drugs, which may reflect differing assay parameters or in vivo pharmacology of each agent. This may be improved in some instances by testing the major metabolic product of the drug, with consideration of an achievable plasma drug concentration. There is increasing recognition of the interplay between tumour cells and the surrounding tumour microenvironment in mediating drug response. The tumour microenvironment includes stromal cancer-associated fibroblasts and immune cells, which may modulate response to therapy via the cross-talk of growth factors and cytokines [66]. These components are absent in standard epithelial organoid cultures, which constitutes a key limitation to predicting patient response.

Initial studies incorporating tumour microenvironment components into organoid cultures have focused on lymphocytes, as cytotoxic CD8+ tumour-infiltrating T-lymphocytes have been shown to play a vital role in anti-tumour immune responses [67]. Autologous lymphocytes may be isolated directly from surgical or endoscopic tumour samples, or from peripheral blood mononuclear cell samples [68,69]. Kong et al. established co-cultures of organoids and tumour-infiltrating lymphocytes (TIL) from biopsies of rectal cancer patients undergoing neoadjuvant chemoradiotherapy [69]. Greater ex vivo tumour cell killing by autologous lymphocytes was observed in co-cultures from patients demonstrating complete pathological response compared with patients demonstrating incomplete response. This suggests that the lymphocyte component of the tumour microenvironment influences the response to chemotherapy and radiotherapy. Therefore, the incorporation of patient-specific TILs into organoid models may improve the prediction of treatment response.

Immune checkpoint inhibitors, which ameliorate the suppression of anti-tumour T-lymphocyte responses, have emerged as an additional line of treatment for CRC. The anti-programmed cell death protein 1 (PD1) monoclonal antibodies nivolumab and pembrolizumab, either alone or in combination with the anti-cytotoxic T-lymphocyte antigen 4 (CTLA-4) monoclonal antibody ipilimumab, are indicated in the setting of mismatch repair deficiency. These CRCs are amenable to immune checkpoint inhibitors due to their high neoantigen burden and immune cell infiltration [10]. However, the majority of CRCs have proficient mismatch repair and do not respond to immune checkpoint inhibitors. Accordingly, in a co-culture study by Chalabi et al., autologous TILs and peripheral blood lymphocytes only responded to tumour organoids derived from patients with deficient mismatch repair CRC [70]. Current research is exploring combinations of immune checkpoint inhibitors and various other targeted therapy and chemotherapy agents, as potential strategies to improve the response of proficient mismatch repair CRCs [71]. Co-cultures of patient-derived tumour organoids and autologous lymphocytes may play a future role in predicting treatment response.

Standard epithelial organoid models also exclude other immune cells which may influence the response to current standard-of-care therapies or provide potential targets for novel immunotherapies. Cytotoxic natural killer (NK) cells contribute to anti-tumour responses. Recent studies have treated CRC organoids with NK cell-conditioned media to measure the induction of apoptosis [72], and co-cultured organoids with a standardised chimeric antigen receptor (CAR)-NK cell line to measure CAR-NK cytotoxicity, which is an important step for modelling CAR efficacy in solid tumours [73]. Neutrophils and macrophages have also been co-cultured with CRC organoids; however, they demonstrated short viability times of 4 h and 48 h, respectively [74], which may limit their inclusion in drug sensitivity studies. Neal et al. described a comprehensive air-liquid interface (ALI) approach whereby patient-derived cancer organoids were extracted with their tumour microenvironment intact [75]. This allowed for the retention of the tissue architecture, and the inclusion of the differentiated immune cell repertoire and cancer-associated fibroblasts. The ALI organoid models were able to capture the activation of TILs in response to immune checkpoint inhibitors. However, ALI cultures demonstrate a progressive loss of their immune cells and fibroblasts over the 1–2 months following establishment, which may limit their feasibility for larger-scale drug sensitivity screens.

A further emerging platform known as “organoid-on-a-chip” involves the culture of organoids on bioengineered flow-through cell chambers that utilise complex microfluidics [76]. In these models, the organoids and their tumour microenvironment are supported by microfluidics that allow for the control of nutrient transfer and waste removal. Developments are in progress to culture functional, microfluidic-perfused vasculature within these models, to support ex vivo testing of anti-angiogenic agents in addition to immunotherapy agents [60,61]. More recently, Ding et al. developed a microfluidics droplet platform to generate patient-derived micro-organospheres (MOS), which preserve the immune cells and fibroblasts of the tumour microenvironment [77]. In this initial proof-of-concept study, MOS’s from CRC biopsies proliferated rapidly to allow for the screening of a panel of 119 FDA-approved compounds within 14 days. Additionally, MOSs from lung cancer biopsies were able to model TIL-induced killing in response to immune checkpoint inhibition. This study highlights a novel and clinically translatable system for realising the potential of ex vivo drug sensitivity testing for precision medicine purposes. With an increasing recognition of the role of the tumour microenvironment in modulating the response to therapy, such developments in organoid technology will likely facilitate the improved modelling of patient response.

## 7. Conclusions

Rising interest in patient-derived tumour organoid models has led to several studies exploring the relationship between ex vivo sensitivity and patient response in CRC. Most studies have focused on radiotherapy and chemotherapy options, providing evidence to support organoid sensitivity testing of ionising radiation, 5FU, and irinotecan, and to a lesser extent, oxaliplatin and TAS-102. One study reported organoid modelling to have a 100% negative predictive value, avoiding unnecessary treatments and associated adverse effects for patients, and ineffectual expenses for the healthcare system [27]. Overall, patient-derived tumour organoids are a promising platform for personalised medicine in the context of standard-of-care treatment options. However, there is a need for the standardisation of tissue sampling, organoid culture, and drug sensitivity testing protocols. Intra-tumoural heterogeneity may impact drug sensitivity, which may be somewhat overcome by increasing the number of spatially separated biopsies used for organoid culture. Drug sensitivity may also be more accurately predicted by sampling tumour metastases, which ultimately are the target of systemic therapies. In the context of novel treatment options, drug access and funding also remain key challenges. As existing studies on identifying tailored novel treatment options have been conducted in the refractory metastatic setting, further prospective trials enrolling patients at earlier stages of treatment are required. An important limitation of patient-derived organoid models is the absence of the complete tumour microenvironment. This prevents the modelling of response to agents that predominantly act on tumour stroma, including anti-angiogenic agents, immunotherapy agents, and some kinase inhibitors. Advancements in culture methods are in development to incorporate various stromal components, which may support ex vivo sensitivity testing of a wider range of treatment options and improve overall modelling of patient response. This technology offers enormous potential to inform therapeutic regimens to improve patient response and reduce adverse events by limiting exposure to options with little predicted efficacy. As the predictive potential of individual agents is confirmed in clinical trials, it will now be necessary to develop standardised, high-throughput, and cost-effective methods for incorporation of tumour organoids in routine pathology practice, to ensure broad access to this transformative technology.

## Figures and Tables

**Table 3:** Summary of studies associating organoid sensitivity and patient response to radiotherapy.

Author, Year [Reference]	(Neo)Adjuvant and/or Metastatic Setting	Radiotherapy
Lv et al., 2022 [37]	Neoadjuvant	*n* = 91
Hsu et al., 2022 [64]	Neoadjuvant and Metastatic	*n* = 13
Park et al., 2021 [65]	Neoadjuvant and Metastatic	*n* = 19
Yao et al., 2020 [24]	Neoadjuvant	*n* = 80
Ganesh et al., 2019 [26]	Neoadjuvant and Metastatic	*n* = 7
	Positive association identified between organoid sensitivity and patient response	
	Potential association between organoid sensitivity and patient response	
	No association between organoid sensitivity and patient response	
	*n* = Number of patients	

## Supplementary Materials

The following supporting information can be downloaded at: https://www.mdpi.com/article/10.3390/cancers15030805/s1, Table S1: Summary of organoid culture medium components used in studies of ex vivo drug sensitivity and patient treatment response, Table S2: Summary of organoid drug sensitivity and radiosensitivity testing methods used in studies of ex vivo drug sensitivity and patient treatment response.
